# Gender-based violence and associated factors among female sex workers in Ethiopia. Evidence from The National Bio-behavioral Survey, 2020

**DOI:** 10.3389/fpubh.2023.1213725

**Published:** 2024-01-31

**Authors:** Lemessa Debel, Jemal Ayalew, Saro Abdella, Jaleta Bulti, Birra Bejiga, Fayiso Bati Wariso, Wudinesh Belete, Abebe Habtesilase, Silesh Lulseged

**Affiliations:** ^1^Ethiopian Public Health Institute, HIV/TB Research Directorate, Addis Ababa, Ethiopia; ^2^Department of Statistics, Wollo University, College of Natural Science, Dessie, Ethiopia; ^3^Addis Ababa University, Faculty of Medicine, College of Health Sciences, Addis Ababa, Ethiopia

**Keywords:** female sex workers, gender-based violence, sexual violence, physical violence, Perpetrators and victims

## Abstract

**Background:**

Gender-based violence (GBV) is usually defined as unequal power relations between men and women, which poses a widespread public health problem. The study evaluated the prevalence and factors associated with GBV among female sex workers (FSWs) in Ethiopia.

**Method:**

We used cross-sectional bio-behavioral data collected using respondent-driven sampling (RDS) in 2020 from 16 towns in Ethiopia. Descriptive statistics was analyzed to summarize the study population characteristics and prevalence of GBV, and a multilevel logistic regression model was applied to identify associated factors for GBV. A *p*-value of ≤0.05 was used as a threshold for statistical significance.

**Result:**

Of 6,085 participants, 28.1% had experienced GBV during the last 12 months, among which 12.7% and 22.3% experienced physical and sexual violence, respectively. FSWs aged 15–24, and 25–34 than those 35 years or more, had a non-paying than paying partners, had 31–60, 61–90, and over 91 than those had less than 30 paying partners, ever had anal sex than those not, condom failure than those not, mobile female sex workers when compared with those not mobile at different town; 3–5 and ≥ 6 years than those less than 3 years stayed in selling sex, street-based, and multiple places selling sex than those used other venues were significantly associated with GBV.

**Conclusion:**

Gender-based violence is a substantial problem among FSWs in Ethiopia, with significant implications for program planning on prevention and response to mitigate the occurrence and impact of GBV among FSWs.

## Background

The United Nations defines GBV as any act of violence that results in or is likely to result in physical, sexual, or psychological harm or suffering to women, including threats of such acts, coercion, or arbitrary deprivation of liberty, whether occurring in public or in private life (1). The definition emphasizes that violence is a manifestation of historically unequal power relations between men and women, which have led to the domination over and discrimination against women by men that causes various health problems.

Female sex workers are at heightened risk for GBV (2, 3). Gender-based violence among female sex workers may cause a widespread public health problem including the acquisition of HIV and other sexually transmitted infections (STIs) through unprotected sex and forced sex, physical injury, and psychological trauma. An increasing body of evidence suggests that sexual violence among FSWs may be associated with individual and interpersonal characteristics and a broad range of environmental factors (4–6).

Reports indicate that the prevalence of GBV at the workplace among FSWs varies from 14 to 54% globally (3, 4, 6). Sexual and/or physical GBV was 75% in Latin America and the Caribbean (7), whereas GBV among mobile FSWs in India was 30.5% (8). Physical violence was reported in 65% of some studies (5, 7, 9). A study from South India indicated that the overall GBV among FSWs was 24%, of which 33% and 7% were exposed to physical and sexual violence, respectively (8). In comparison, sexual violence was reported to be 16.9% in Iran (9). Different reports from five Southern African countries show that physical and sexual violence among FSWs in the past 12 months was as high as 70% (10). This figure was 87%, 82%, and 40% in Kenya, Uganda, and Tanzania, respectively (11–13). In Ethiopia, the studies conducted in specific areas indicated that the overall workplace sexual violence was 28%, and the highest prevalence of 75.6% was reported at Mekele town among this population group (14, 15).

Female sex workers use different settings and venues for selling sex where GBV could be committed. The most common settings reported include homes, workplaces such as bars, and on the street (5, 16). The perpetrators of GBV vary by their type of relationship and setting, which was approximately 63.8% with clients in the Caribbean and 18.7% with brothel management in Nigeria (17). The other different factors such as migration to meet new clients (18, 19), stigma, discrimination, or harassment, and changes in work environments increase the exposure of FSWs to GBV (18–21).

Indeed, the majority of these studies identified that individual-level risk factors associated with physical or sexual violence among FSWs were limited to small geographic areas and did not take into account interpersonal and environmental factors beyond individual control that may play significant roles in GBV. Therefore, this study aimed to determine the magnitude of GBV and associated factors related to individual, interpersonal, and environmental factors by considering the cluster effect across towns in Ethiopia.

## Materials and methods

A cross-sectional survey using a respondent-driven sampling process was conducted from 2019 to 2020 to identify the prevalence of HIV and other STIs, and GBV among FSWs in 16 cities and towns of Ethiopia. The study population was FSWs who had had sex for money or goods in the capital cities (Addis Ababa, Hawassa, Bahir Dar, Harar, Gambella, and Dire Dawa) of six administrative regions and ten sub-national major towns (Adama, Jimma, Nekemte, and Shashemane; Arba Minch, Dilla, and Mizan; Gondar and Kombolcha; and Logia), which could represent the population groups in Ethiopia.

### Sampling procedures

The sample size was determined by a single population proportion formula, assuming a 95% confidence interval, *α* = 0.05, margin of error (*d*) = 35%, and proportion (*p*) = 2% (22), and a design effect of 1.5 with a replacement for non-responders was used. The minimum desired sample size of FSWs in the 6 regional capitals and 10 major towns with a 10% contingency was 6,085 FSWs. This was allocated to the 16 sites proportionate to population size (Table 1).

**Table 1:** Sample sizes and seed distribution by study sites and the National HIV and Other STI Bio-Behavioral Survey, Ethiopia, 2019–2020 (*N* = 6,085).

Study site	Sample size	Seeds	Study site	Sample size	Seeds
Addis Ababa	1101	13	Dilla	251	5
Jimma	254	5	Dire Dawa	434	5
Adama	676	8	Logia/Semera	251	5
Arba Minch	251	5	Bahir Dar	372	8
Hawassa	522	8	Gonder	250	5
Kombolcha/ Dessie	251	5	Nekemte	257	5
Gambella	468	6	Shashemane	250	5
Mizan	255	5	Harar	242	5
Total				6085	98

The participants were included in the study by respondent-driven sampling (RDS) method. The RDS technique was started by identifying seeds or initial participants. The “seeds” were selected based on the type of sex worker, age category, and geographic location. The number of seeds for each site was determined based on the result of the formative assessment. Accordingly, five seeds for each site that had less than 450 sample sizes, 6–8 seeds for each site that had between 450 and 900 sample sizes, and 12 for 1,250 sample sizes were recruited. Then, FSWs with a known social network were given three coupons so that they could invite their friends or other FSW contacts that were in their network. The coupon was active from the day the coupon was given by the potential recruiter and expired after 2 weeks or when the study was completed. Coupons that were damaged, not readable, photocopied, had no seal/stamp on them, or were not the original ones were declared not valid to avoid desirability bias. Each participant who visited the study site brought the coupon that was identified by number and by who referred them. Based on these criteria, each new participant was given coupons and asked to recruit three additional acquaintances. This process continued until the desired sample size was achieved and the RDS equilibrium was attained. The respondents gave their informed consent to participate in the study. A total of 98 seeds were used for the enrollment of a total of 6,085 FSWs from the 16 study sites, and all 6,085 participated in the study. The majority of the study participants were recruited from seeds that generated two to six waves. A maximum of 16 waves were attained.

### Inclusion criteria

Across all 16 study sites, participants were eligible for the study if they were 15 years or older, reported having sex in exchange for money, goods, services, or drugs with more than one client within 12 months before the interviews, and lived or worked in the city/town where the study was conducted.

### Definitions of terms

**Khat** is a shrub (*Catha edulis*) of the staff-tree family that is cultivated in the Middle East and Africa for its leaves and buds which are the source of a habituating stimulant when chewed or used as a tea (23).

**Condom failure** is the situation when the condom tears off or partially or completely slips off the penis or is removed from the penis during sexual intercourse (24).

### Study variables

The outcome (dependent) variable was GBV experienced by FSWs. The independent variables were individual [(**socio-demographic factors**: age, educational status, marital status, income, and residence), alcohol consumption, chewing khat, and duration since engagement in sex work], **interpersonal with partners** (number and type of partner, ever had anal sex, and condom failure), and **environmental** (change of place in the last 6 months, number of cities FSWs practiced selling sex and practiced venue, FSWs’ mobility, and place of sex work).

### Data collection

A standard National HIV and other STIs Bio-Behavioral Survey (NHSBS) questionnaire includes questions on demographic characteristics, history of sexual and other risk behaviors, and GBV (25). A response plan was made to refer FSWs who reported recent GBV victims to the concerned body to get the necessary counseling and support services. The data were collected using the Open Data Kit (ODK) software on a tablet.

### Data analysis

The RDS recruitment process (tree of recruitment), evaluation of the RDS assumptions, and generation of weights were all done using R statistical software. The entire dataset was combined with the RDS weights using the RDS-II function before being exported for additional analysis. The frequency in their raw, median, inter-quartile range (IQR), and RDS-adjusted forms was computed using STATA software. The ODK software’s data were exported to MS Excel, cleaned up, and then imported into STATA Verssion16 for analysis.

Owing to the different geographic areas, GBV prevalence and the associated factors could be influenced by the differences in site (city/town)-level factors. A multilevel logistic regression analysis was considered to assess for variation by town and identify their association with the independent variables. This analysis used a two-level multilevel fixed-effect logistic regression model. Level 1 variables were all the independent variables categorized as individual-level variables and level 2 variables were the towns. To measure the impact of the level 2 variables (town) in the multilevel regression model, we used intra-class correlation (ICC), and the between-town variation accounted for a portion of the total variation in the response variable. The effects of individual-level predictors were quantified by the estimates from the fixed-effect part of the model with a *p*-value less than 0.05 at 95% CI that did not include one.

### Ethical considerations

The study protocol and procedures were approved by the Research Ethics Committee of the Ethiopian Public Health Institute (Ethics reference number: EPHI-IRB-108-2018). FSWs were invited to participate in the study after receiving a brief introduction to the aims of the study and the potential risks and benefits of participating in the study. Those who provided verbal informed consent were interviewed one-on-one in a private room by trained interviewers. The collected data were kept confidentially and secured in the Ethiopian Public Health Institute’s database.

## Results

### Socio-demographic and other related characteristics

In total, 6,085 FSWs were recruited from the 16 survey sites. The median age of participants was 25.0 IQR of ±8 years [22, 30]. More than four-fifths (82.7%) of them were literate (attended primary school and above). A majority (85%) consumed alcohol, and approximately 62.9% of them chewed *Catha edulis* (khat) (Table 1). Nearly two-thirds (61.5%) of them had up to 60 paying partners in the last 6 months, and most (81%) stayed in the same town in the past 12 months before the survey (Table 2).

**Table 2:** Socio-demographics and other related characteristics of female sex workers in Ethiopia (*N* = 6,085).

Variables	Frequency	%
Age		
15–24	2,595	42.7
25–34	2,671	43.9
≥ 35	819	13.5
Educational status		
No formal education	1,054	17.3
Primary school	3,560	58.5
Secondary school and above	1,471	24.2
Income due to selling sex/month in dollars		
< 86 $	1778	29.2
86–171.99 $	2066	34.0
>172–259 $	1,175	19.3
≥ 259 $	1,066	17.5
*Catha edulis* (khat) chewing		
No	2,258	37.1
Yes	3,827	62.9
Alcohol and drug use		
No	915	15.0
Yes	5,170	85.0
Currently, do you have a regular non-paying partner?		
No	4,347	71.4
Yes	1738	28.6
Number of non-paying partners in the last 6 months		
Never	4,347	71.4
Only one	1,404	23.1
2 and more	334	5.5
Number of paying partners in the last months		
4–30	2,308	37.9
31–60	1,436	23.6
61–90	696	11.4
≥ 91	1,645	27.0
Ever had anal sex		
No	5,659	93.0
Yes	426	7.0
Condom failure		
No	4,260	70.0
Yes	1825	30.0
Condom Use		
Consistent	5,119	84.1
Inconsistent	966	15.9
FSWs** movement/change of place in 6 months		
No	4,572	75.1
Yes	1,513	24.9
Number of cities where the FSWs moved and practiced selling sex		
One town	4,933	81.1
Two town	776	12.8
Three or more towns	374	6.1
Years lived in current city/place		
< 5	2,308	37.9
5–10	1,559	25.6
≥ 11	2,218	36.5
Number of years as sex worker		
< 3	2,343	38.5
3–5	2,202	36.2
≥ 6	1,537	25.3
Venue		
Bar/Hotel	1,132	18.6
Street	1,221	20.1
Multiple	3,040	49.9
Others	692	11.4

FSWs** movement/change place is when FSWs change their place of work from town to town to meet more clients or their clients.

### Prevalence of types of gender-based violence

Among the 6,085 FSWs aged 15 years and above who participated in the study, over a quarter (1710) [28.1%; 95% CI (26.99, 29.25)] had experienced physical or sexual violence or both types in the last 12 months before the survey, of which 1,354 [22.3%; 95% CI (21.23, 23.31)] and 771 [12.7%; 95% CI (11.86, 13.53)] experienced physical violence and sexual violence, respectively (Table 3).

**Table 3:** Prevalence and types of violence among female sex workers in Ethiopia (*N* = 6,085).

Characteristics	Categories	(*n*)	% (95% CI)
Violence among female sex workers in the last 12 months before the survey	Yes	1710	28.1 (26.99, 29.25)
	No	4,375	71.9 (70.75, 73.01)
Physical violence in the last 12 months before the survey	Yes	1,354	22.3 (21.23, 23.31)
	No	4,731	77.8 (76.89, 78.78)
Sexual violence in the last 12 months before the survey	Yes	771	12.7 (11.86, 13.53)
	No	5,314	87.3 (86.47, 88.14)

### Gender-based violence perpetrators and victims reported to the police

**Figure 1:**
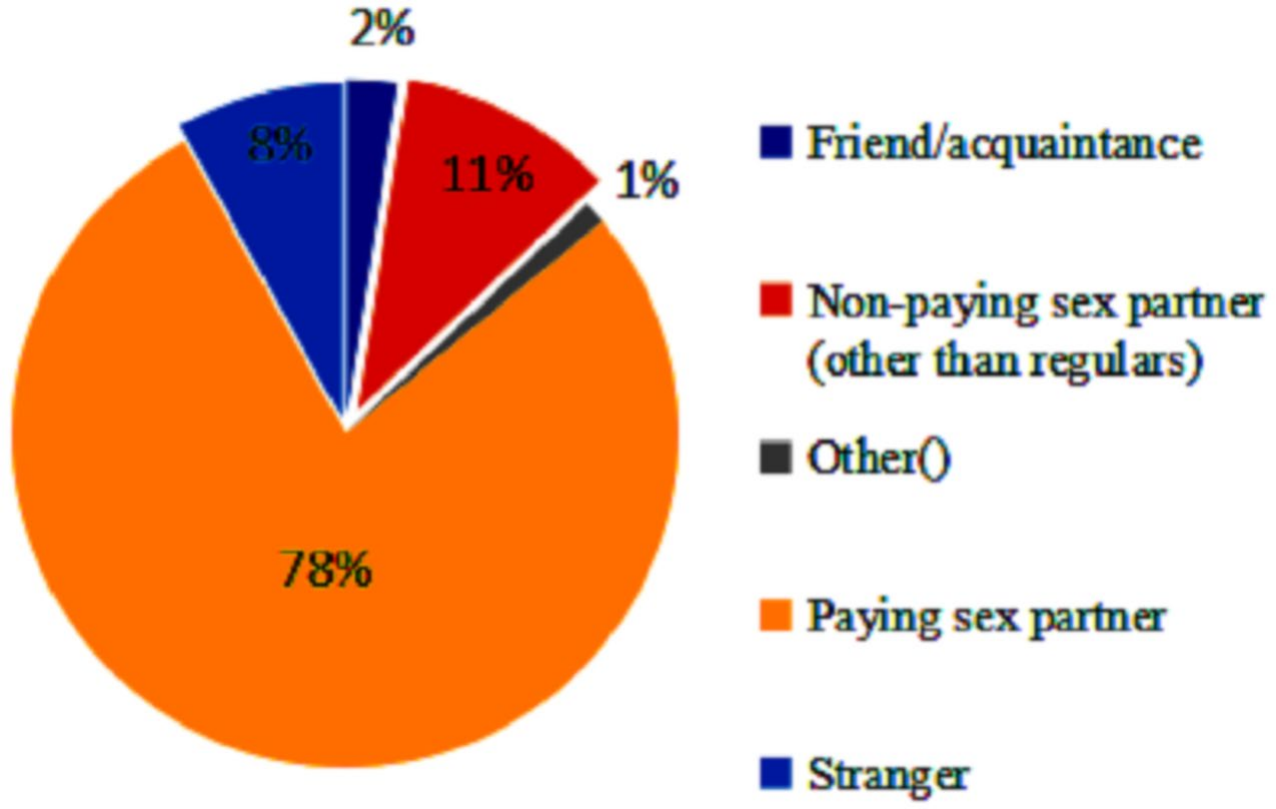
Perpetrators of violence.

**Figure 2:**
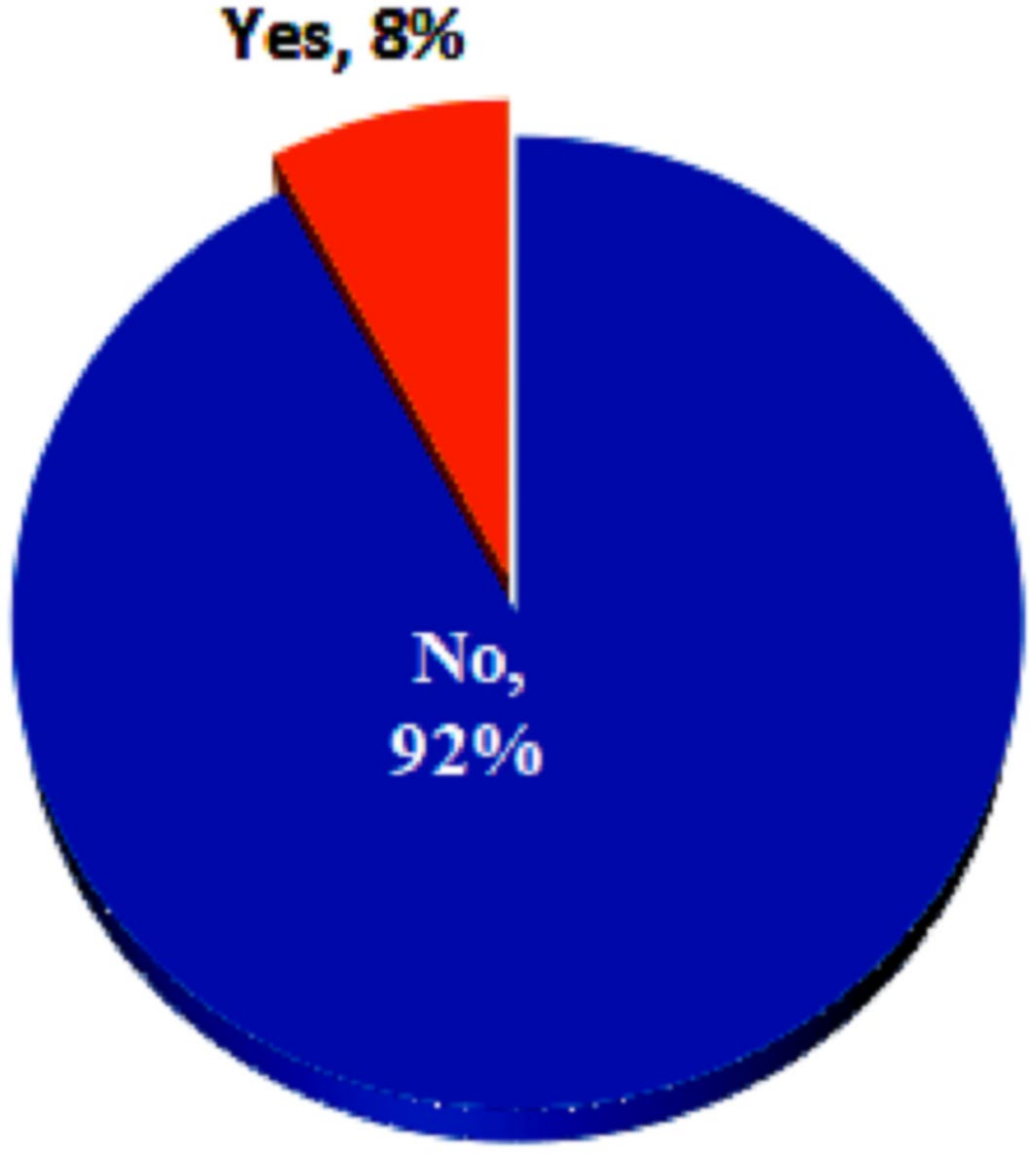
Victims reported to the police.

Nearly four-fifth (77.9%) of the perpetrators who committed GBV were paying partners followed by non-paying partners other than regular non-paying partners (10.5%) (Figure 1). Only 20% of the victims reported to the police (Figure 2).

### Gender-based violence and associated factors

The results on the multilevel fixed-effect logistic regression model diagnosis show that the model fitted well for the data over the standard logistic regression to assess determinants for GBV ( $X^{2}=174.66,\;p<0.001$ ). The results of the ICC of the two multilevel models revealed that approximately 8.0% of the variations in the likelihood of GBV were explained by the variation among the towns in Ethiopia. As we can see from the 95% CI [0.04, 0.15], the variation among the towns was statistically significant, and hence any estimation without considering this effect will result in a biased estimate (Table 4).

**Table 4:** Bivariate and multivariate logistic regression analyses of gender-based violence and associated factors among female sex workers.

Characteristics	Not violated	Violated	COR (95% CI)	*p*-value	AOR (95% CI)		*p*-value
Age							
15–24	1891 (73)	701 (27)	1.36 (1.11, 1.65)	0.002	1.35 (1.07, 1.69)		0.010**
25–34	1844 (69.2)	822 (30.8)	1.58 (1.31, 1.91)	<0.001*	1.44 (1.20, 1.81)		<0.001**
35+	635 (77.9)	180 (22.1)	1		1		
Educational status							
Not attended school	754 (71.7)	297 (28.3)	1.14 (0.9. 1.14)	0.268			
Primary school	2,551 (71.7)	1,005 (28.3)	1.04 (0.86, 1.25)	0.685			
Secondary and above	1,065 (72.6)	401 (27.4)	1				
Income from selling sex							
< 86 $	1,359 (76.8)	411 (23.2)	1.16 (0.99, 1.36)	0.059			
86–172 $	1,496 (72.5)	567 (27.5)	1.37 (1.14, 1.64)	0.001*			
172.4–259 $	812 (69.1)	363 (30.9)	1.34 (1.12, 1.63)	0.002*			
≥ 259 $	703 (66)	362 (34)	1				
*Catha edulis* (khat) chewing							
No	1794 (79.6)	459 (20.4)	1				
Yes	2,576 (67.4)	1,244 (32.6)	1.78 (1.47, 2.17)	< 0.001*			
Alcohol use							
No	725 (79.6)	186 (20.4)	1		1		
Yes	3,645 (70.6)	1,517 (29.4)	1.42 (1.19, 1.69)	<0.001*	1.17 (0.97, 1.41)		0.103
Had non-paying partner in the last 6 months							
No	3,300 (76.1)	1,038 (23.9)	1		1		
Yes	1,070 (61.7)	665 (38.3)	1.84 (1.62, 2.09)	<0.001*	1.58 (1.38, 1.80)		<0.001**
Number of paying partners in the last 6 months							
≤30	1775 (77.1)	527 (22.9)	1		1		
31–60	1,028 (71.7)	405 (28.3)	1.32 (1.12, 1.56)	0.001	1.24 (1.04, 1.47)		0.016**
61-90	482 (69.3)	214 (30.7)	1.42 (1.15, 1.74)	0.001	1.32 (1.07, 1.64)		0.011
≥ 91	1,085 (66.1)	557 (33.9)	1.74 (1.47, 2.05)	<0.001*	1.48 (1.24,)1.77		<0.001**
Ever had anal sex							
No	4,168 (73.8)	1,479 (26.2)	1		1		
Yes	202 (47.4)	224 (52.6)	2.77 (2.25, 3.41)	<0.001*	2.05 (1.64, 2.55)		<0.001**
Condom failure							
No	3,326 (78.2)	927 (21.8)	1		1		
Yes	1,044 (57.4)	776 (42.6)	2.62 (2.32, 2.97)	<0.001*	2.33 (2.05, 2.65)		<0.001**
FSWs’ change of place in the last 6 months							
No	3,427 (75.2)	1,133 (24.8)	1			1	
Yes	943 (62.3)	570 (37.7)	1.74 (1.53, 1.99)	<0.001*	1.39 (1.20, 1.60)		<0.001**
Number of cities/towns FSWs practiced selling sex							
One town	3,691 (75.0)	1,230 (25.0)	1		1		
Two towns	469 (60.4)	307 (39.6)	1.87 (1.58, 2.20)	<0.001*	1.46 (1.23, 1.75)		<0.001**
Three or more towns	208 (55.6)	166 (44.4)	2.04 (1.62, 2.58)	<0.001*	1.42 (1.11, 1.82)		0.006**
Number of years as a sex worker							
Less than 3 years	976 (71.6)	388 (28.4)	1		1		
4+ years	3,394 (72.1)	1,315 (27.9)	1.43 (1.23, 1.66)	<0.001	1.34 (1.15, 1.75)		0.002**
Venue							
Bar/Hotel	808 (71.7)	319 (28.3)	1		1		
Street	958 (78.6)	261 (21.4)	1.37 (1.12, 1.68)	0.002	1.42 (1.28, 1.83)		<0.001**
Multiple	2056 (67.7)	980 (32.3)	1.59 (1.35, 1.90)	<0.001	1.53 (1.29, 1.86)		0.001**
Others	548 (79.3)	143 (20.7)	0.88 (0.69, 1.13)	0.313	0.92 (0.71, 1.19)		0.524
Random effects							
**Towns/cities**					0.27 (0.13, 0.57)		
ICC^**^					0.08 (0.04, 0.15)		
LR test^**^vs. logistic model							
**Chibar2 = 174.66** ^**^							
***p*-value = 0.000**							

**—shows variables shown significant association at the stated p-value.

The fixed effect of the multilevel logistic regression analysis indicated that age groups 5–24 and 25–34 years [AOR = 1.35; 95% (1.07, 1.69); *p* = 0.01] and [AOR = 1.44; 95% CI (1.20, 1.81); *p* < 0.001] than age group 35 years or more have shown an association with GBV among FSWs. Concerning partners’ characteristics; having a non-paying partner [AOR = 1.58; 95% CI (1.38, 1.80); *p* < 0.001] than paying; number of paying partners having 31–60, 61–90; and ≥ 91 which were [AOR = 1.24; 95% CI (1.04, 1.47); *p* = 0.016], [AOR = 1.32; 95% CI (1.07, 1.64); *p* = 0.011)], and [AOR = 1.48; 95% CI (1.24, 1.77); *p* < 0.001] than with less than 30, respectively, have shown association with GBV among FSWs. It also indicated that the trend of GBV among FSWs increased with an increase in the number of paying partners. Related to sexual act, those FSWs who ever had on the other side, sexual acts ever had anal sex [AOR = 2.05; 95% CI (1.64, 2.55); *p* < 0.001] than those not; condom failure [AOR = 2.33; 95% CI (2.05, 2.65); *p* < 0.001] than those not, and number of years 3–5, and 6 years or more [AOR = 1.21; 95% CI (1.05, 1.41)] and [AOR = 1.34; 95% CI (1.15, 1.75); *p* = 0.002) than those stayed less than 3 years in selling sex, respectively, were associated with FSWs GBV.

Regarding the environment in which FSWs reside and sell sex; changing place or mobile than those not in the last 6 months [AOR = 1.39; 95% CI (1.20, 1.60); *p* < 0.001]; the number of cities two and three or more FSWs stayed and selling sex [AOR = 1.46; 95% CI (1.23, 1.75); *p* < 0.00] and [AOR = 1.42; 95% CI (1.11, 1.82); *p =* 0.006], respectively, than those stayed at one town/city, sex work in the street and multiple places [AOR = 1.42; 95% CI (1.28, 1.83); *p* < 0.001] and [AOR = 1.53 95% CI (1.29, 1.86); *p* = 0.001] than those use other venue, respectively, were associated with GBV among FSWs.

## Discussion

The overall prevalence of GBV experienced among FSWs in our study was 28.1%, which falls within the range of globally reported GBV prevalence among this population group (4). The prevalence of GBV among FSWs by our study was approximately a third of that reported by studies conducted in Mombasa, Kampala, and Mekele (11, 12, 15) and less than half of that reported from Latin America and the Caribbean, Nigeria, Zambia, and Tanzania (7, 12, 13, 17). On the other hand, our finding was almost similar to what had been reported from south India (30.5%) and the finding from a meta-analysis of isolated studies in Ethiopia (16), while it was higher than what was reported by a study in Iran and the Middle East (6). These variations could be due to the variations in sociocultural differences between the countries and/or the continents.

The physical violence and the sexual violence among FSWs in our study were 22.3% and 12.8%, respectively. The prevalence of physical violence among FSWs was similar to what had been reported by the study conducted in Mombasa, Kenya, but was two times more than the finding from South India (8, 11), three times more than the prevalence reported by Tanzania (13), and two times less than what was reported by the study from Nigeria (17). On the other hand, sexual violence in our study showed a similar finding to the study conducted in South India among mobile FSWs but three times less than the findings from Nigeria and Tanzania and five times less than what was reported from North Karnataka in South India (9, 13, 17).

The wide variation in the prevalence and type of gender-based violence among FSWs across countries indicates specific individual characteristics and contextual factors need to be understood to design prevention and response interventions. Among the FSWs in the study, we documented that age, having non-paying partners and number of paying partners in the 6 months preceding data collection, having ever had anal sex, mobility of FSWs, number of towns where they practiced selling sex, number of years the FSWs stayed in selling sex, and the venue at which selling sex was practiced were associated with violence.

Our finding on prevalence and predictors of sexual violence among FSWs concurs with the report from a study conducted in Northern Ethiopia, which showed that FSWs in the younger age group had a higher rate of violence than the older age category (15). In contrast, a study in Nigeria identified more violence among older FSWs than the younger age group (17). This might be explained as the older age group FSWs could have more awareness of it and experience in escaping from perpetrators, while the differences could be due to sociocultural and other drivers of GBV among FSWs in the two countries. Among other factors associated with FSWs, GBV in our study was having non-paying partners in the last 6 months. This was similar to the findings from North Karnataka in South India, which examined determinants of GBV among FSWs in an intimate partner relationship (9). Similar to the study conducted in Zambia, our study showed that the number of paying partners in the last 6 months was associated with GBV among FSWs, while having more than nine paying partner by our study has half less GBV among FSWs than the finding from Zambia (12).

We also found that FSWs who ever practiced anal sex experienced GBV more often than their counterparts, which was similar to what was reported by the African Sex Work Alliance among FSWs in Nairobi but two times more than that was reported by the study conducted at Kampala (12). Regarding FSW mobility and the number of cities and towns where they practiced selling sex in association with GBV, the finding from our study was similar to the findings from the study conducted in India and Northern Vietnam (20, 21).

This study also identified an association between GBV and the number of years of selling sex (more than 3 years) sex by FSWs. This finding corroborated the similarity with the finding from a study conducted in Northern Ethiopia (19). Concurring with the findings from the Ethiopia National Survey on FSWs (16), the venue type where the FSWs used to sell sex was significantly associated with violence in our study. This indicated that there are still similarities in trends of venues where FSWs used to pickup their partners. Unlike previous studies (14–16), other socio-demographic and individual factors including educational status, income, and chewing *Catha edulis* (khat) were not associated with GBV among FSWSs in our study.

## Limitations

As the findings from this study relied on self-reported measures, there might be social desirability and recall biases. These need to be considered in the interpretation of the results as well. The strength of the study was the nationally representative number of FSWs from cities and towns that participated, the respondent-driven sampling method (RDS) including eligible participants, and the multilevel logistic regression analysis that was applied to manage the individual level and intra-class correlation (ICC) between-town variation.

## Conclusion and recommendation

The findings from this study revealed that violence among FSWs is high in Ethiopia. It also indicated that GBV among FSWs in Ethiopia is associated with socio-demographic factors, partner characteristics, and the environment where they practice sex selling. The study results have important implications for policy and program planning, prevention, and response to mitigate the occurrence and impact of GBV among FSWs.

## Data availability statement

The raw data supporting the conclusions of this article will be made available by the authors, without undue reservation.

## Ethics statement

The studies involving humans were approved by Ethiopian Public Health Institute IRB. The studies were conducted in accordance with the local legislation and institutional requirements. The participants provided their written informed consent to participate in this study.

## Author contributions

LN developed the concept of the study and contributed to the analysis, manuscript development, and submission. JA contributed to data analysis. SA was involved in manuscript development and review. JB, BB, FW, WB, and AH participated in manuscript development. SL carried out the manuscript review. All authors contributed to the article and approved the submitted version.
